# Principal Components Analysis Using Data Collected From Healthy Individuals on Two Robotic Assessment Platforms Yields Similar Behavioral Patterns

**DOI:** 10.3389/fnhum.2021.652201

**Published:** 2021-05-06

**Authors:** Michael D. Wood, Leif E. R. Simmatis, Jill A. Jacobson, Sean P. Dukelow, J. Gordon Boyd, Stephen H. Scott

**Affiliations:** ^1^Department of Anesthesiology, Pharmacology & Therapeutics, University of British Columbia, Vancouver, BC, Canada; ^2^Toronto Rehabilitation Institute, University Health Network, Toronto, ON, Canada; ^3^Department of Psychology, Queen’s University, Kingston, ON, Canada; ^4^Department of Clinical Neurosciences, University of Calgary, Calgary, AB, Canada; ^5^Centre for Neuroscience Studies, Queen’s University, Kingston, ON, Canada; ^6^Department of Medicine, Queen’s University, Kingston, ON, Canada; ^7^Department of Critical Care Medicine, Queen’s University, Kingston, ON, Canada; ^8^Department of Biomedical and Molecular Sciences, Queen’s University, Kingston, ON, Canada

**Keywords:** Robotics, behavior, motor system, principal components analysis, agreement

## Abstract

**Background:**

Kinarm Standard Tests (KSTs) is a suite of upper limb tasks to assess sensory, motor, and cognitive functions, which produces granular performance data that reflect spatial and temporal aspects of behavior (>100 variables per individual). We have previously used principal component analysis (PCA) to reduce the dimensionality of multivariate data using the Kinarm End-Point Lab (EP). Here, we performed PCA using data from the Kinarm Exoskeleton Lab (EXO), and determined agreement of PCA results across EP and EXO platforms in healthy participants. We additionally examined whether further dimensionality reduction was possible by using PCA across behavioral tasks.

**Methods:**

Healthy participants were assessed using the Kinarm EXO (*N* = 469) and EP (*N* = 170–200). Four behavioral tasks (six assessments in total) were performed that quantified arm sensory and motor function, including position sense [Arm Position Matching (APM)] and three motor tasks [Visually Guided Reaching (VGR), Object Hit (OH), and Object Hit and Avoid (OHA)]. The number of components to include per task was determined from scree plots and parallel analysis, and rotation type (orthogonal vs. oblique) was decided on a per-task basis. To assess agreement, we compared principal components (PCs) across platforms using distance correlation. We additionally considered inter-task interactions in EXO data by performing PCA across all six behavioral assessments.

**Results:**

By applying PCA on a per task basis to data collected using the EXO, the number of behavioral parameters were substantially reduced by 58–75% while accounting for 76–87% of the variance. These results compared well to the EP analysis, and we found good-to-excellent agreement values (0.75–0.99) between PCs from the EXO and those from the EP. Finally, we were able to reduce the dimensionality of the EXO data across tasks down to 16 components out of a total of 76 behavioral parameters, which represents a reduction of 79% while accounting for 73% of the total variance.

**Conclusion:**

PCA of Kinarm robotic assessment appears to capture similar relationships between kinematic features in healthy individuals and is agnostic to the robotic platform used for collection. Further work is needed to investigate the use of PCA-based data reduction for the characterization of neurological deficits in clinical populations.

## Introduction

Robotic assessment tools can capture granular and high-dimensional kinematic information from the upper limbs that would be very difficult to measure with a human observer, thus potentially facilitating both assessment and rehabilitation (Wu et al., 2013; Colombo et al., 2014; Pila et al., 2017; Veerbeek et al., 2017). Kinarm is one such device, and provides objective parameters of motor, sensory, and cognitive function using the participants’ arms. It has been validated across multiple patient groups (Coderre et al., 2010; Dukelow et al., 2010, 2012; Debert et al., 2012; Simmatis et al., 2017; Gaprielian et al., 2019). Kinarm Standard Tests^TM^ (KSTs) consist of several behavioral tasks that include automated data analysis routines, and which generate up to 20 performance items per task that describe spatial and temporal aspects of performance. However, the breadth of behavioral tasks results in complex datasets being produced (>100 parameters per participant), which may impede meaningful interpretation of data – particularly for those unfamiliar with the meaning or neuroscientific importance of each metric. Therefore, this technology may benefit from the application of data reduction techniques.

Principal component analysis (PCA) is a statistical procedure that is commonly used for dimensionality reduction, which preserves the maximal amount of variance in a given dataset (Pearson, 1901; Hotelling, 1933; Ringnér, 2008). The first principal component (PC) is a linear combination of variables that accounts for the largest amount of variance, followed by the second and so forth (Jolliffe and Cadima, 2016). Our previous study examined PCA for healthy participant’s data. We found that data from the Kinarm End-Point Lab (EP) could be substantially reduced while accounting for a large amount of variance, and that many PCs were comprised of task parameters with high loadings and minimal cross loadings (i.e., items that loaded substantially across multiple components) (Wood et al., 2018).

Kinarm Standard Tests can also be completed on a second robotic platform called the Kinarm EXO^1^. The EXO robots are attached to the arm using troughs and the entire arm is constrained to move in the horizontal plane. In contrast, the EP robots are grasped in each hand permitting horizontal movements of the manipulanda, but with the plane of the arm being largely vertical (i.e., elbows pointing down with the wrist halfway between pronation and supination). These differences in arm geometry could potentially influence motor strategies and the structure of motor performance, even for similar behavioral goals (Beer et al., 2004). As such, it is important to validate results from reduction techniques performed on data from these different Kinarm platforms to ensure that they can produce findings in a manner that is agnostic to the technology used to collect movement data.

Previous studies have explored the use of PCA or exploratory factor analysis with other robotic platforms (Gilliaux et al., 2014; Longhi et al., 2016; Panarese et al., 2016) and others have employed PCA as a dimensionality reduction tool for analyzing motor synergies (Ozturk et al., 2016; Wang et al., 2020). However, some studies reporting PCA of behavioral data have relied on small sample sizes (typically *N* < 50) that could limit generalizability to other samples of participants. Other previous work has explored the use of non-variance-based dimensionality reduction measures to create aggregate representations of Kinarm data (Kenzie et al., 2017); however, this has the limitation of being difficult to relate back to the underlying variables that contribute to the summary statistic that is produced. Our central objective was to replicate PCA across robotic platforms to understand how similar the groupings of kinematic features are across data collection systems with varying arm geometries. We did this by quantifying the agreement between PCA results derived from the EP [published previously (Wood et al., 2018)] and the current Kinarm EXO analysis. Further, the large size of the EXO dataset permitted an examination of whether further data reduction was feasible across behavioral tasks. As such, comparable data reduction results across platforms, and tasks, would suggest similar strategies or features of motor execution that are common across behaviors.

## Materials and Methods

### Participant Recruitment

Healthy participants were community-based and were recruited via advertisements on lab and departmental websites, in local classifieds (online and print), and by word-of-mouth. Trained research staff screened each adult participant (≥18 years old) to ensure that task instructions could be easily understood, and that participants had no prior neurological deficits or medical conditions that could affect upper limb function. Once enrolled, participants were then assessed by our research staff at one of four sites in Canada, which included three sites in Kingston, ON (Providence Care Hospital, St. Mary’s of The Lake Hospital, and Kingston General Hospital) and one in Calgary, AB (Foothills Medical Centre). Participants’ handedness was determined using the Modified Edinburgh Handedness Inventory (Oldfield, 1971). The Queen’s University and Affiliated Hospitals Health Sciences Research Ethics Board and The University of Calgary Conjoint Health Research Ethics Board approved participants’ recruitment and assessment. Informed consent was obtained from each participant prior to the Kinarm assessment. Participants were recruited separately for studies conducted using the EP and EXO platforms. Note that the EP platform is newer and therefore the number of participants who completed each of the tasks presented here is lower and more variable across tasks than with the EXO.

### Robotic Assessment

**FIGURE 1:**
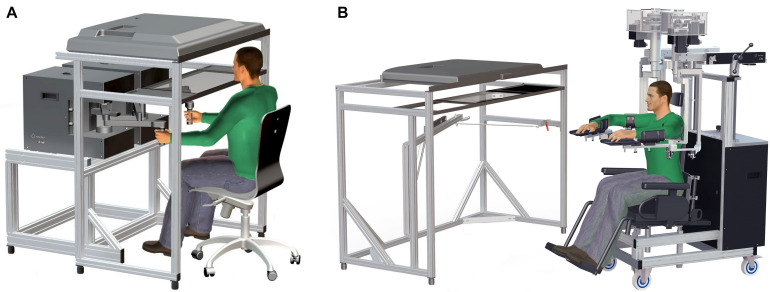
**(A)** Kinarm End-Point Lab with graspable robots and virtual reality system aligned with the horizontal workspace. **(B)** Kinarm Exoskeleton Lab with adjustable robots attached to the arms maintaining arm motion in the horizontal plane and a similar virtual reality system.

Participants were either seated in a height-adjustable chair that was locked in place in front of the EP robotic system (Figure 1A) or were seated in the height-adjustable wheelchair seat when using the EXO robotic system (Figure 1B; Kinarm, Kingston, ON, Canada). For the former, participants were instructed to grasp the EP robotic handles with each hand, permitting free movement of the hands in the horizontal plane with the arm itself in the vertical plane (i.e., elbow largely pointing downward). For the latter, the EXO seat height was adjusted for each participant to achieve shoulder abduction (∼85°) to provide comfort and anti-gravity support of the upper arm and forearm/hand. Thus, movements in the workspace maintained the hand, elbow, and shoulder in the horizontal plane. Participants were seated in the EP or EXO in front of a virtual reality system that displayed each task in the horizontal workspace, and vision of the participant’s arms and hands were occluded with a physical barrier. The participant’s head was positioned in the front center of the display case and visual feedback of actual hand position (when provided) was typically represented on the screen by a white circle (radius = 0.5 cm) in the middle of the handle grasped by the participant (EP) or over their index fingertip (EXO). A trained operator provided instructions from a standardized script prior to starting each task.

The Arm Position Matching (APM) (Dukelow et al., 2010; Mochizuki et al., 2019) task assessed upper-limb position sense (an element of proprioception), whereas the other three tasks broadly assessed upper-limb motor behavior: Visually Guided Reaching (VGR) (Coderre et al., 2010), Object Hit (OH) (Tyryshkin et al., 2014), and Object Hit and Avoid (OHA) (Bourke et al., 2016). Detailed descriptions of all tasks have been reported previously (Coderre et al., 2010; Dukelow et al., 2010; Tyryshkin et al., 2013; Bourke et al., 2016; Wood et al., 2018), but are summarized in Table 1 for reference. Task parameter descriptions can be obtained from Kinarm (see text footnote 1). Within each task, 9–20 performance parameters were produced (parameters analyzed were the same across both robotic platforms), and completing all 4 tasks took < half an hour on a given robotic platform. Note that, in this study, VGR and APM data were analyzed from each of the dominant- and non-dominant arms separately, and so we report 6 total behavioral assessments (i.e., OH, OHA, two separate scores for VGR, and two separate scores for APM, totaling 6). To prevent potential handedness confounds, mixed handedness participants (*n* = 2) were removed from the analysis, and we used performance parameters from both the dominant and non-dominant limbs for two of the tasks (i.e., VGR and APM).

**TABLE 1:** Kinarm behavioral battery task descriptions.

**Task**	**Description**
Arm Position Matching	The robot moves one of the participant’s arms (the “active” arm), and the goal for the participant was to mirror-match the position using their contralateral arm. The participant could not see where their arms were, and as such they had to complete the task “by feel” (i.e., using proprioception).
Visually Guided Reaching	Participants were instructed to perform center out and back reaches to a series of visual targets, starting from a central target. The task instructions specified that the reaches be both quick and accurate.
Object Hit	Virtual objects “fell” toward the participant from the top of the screen, and participants had to hit them away using virtual paddles – one controlled by each hand. Objects fell faster as the task progressed, although the task lasted for a fixed amount of time (∼2.25 min). The objective was to hit as many objects as possible.
Object Hit and Avoid	This task was very similar to Object Hit, except that some of the objects that fell toward the participant were distractors to be avoided. Altogether, eight different object types fell toward the participant, but only two were targets to be aimed for; the other distractors counted against the participant if they were hit by mistake. The objective of this task was to hit as many targets, and as few distractors, as possible.

Automated data collection and analysis software was used to measure arm movements and quantify task performance. Task parameter values for the healthy cohort (also reported in this study) were standardized (mean subtracted and divided by the standard deviation), and then used to create a Box-Cox transform (Box and Cox, 1964), which converted the distribution of standardized scores into a standard Normal distribution (i.e., mean = 0, standard deviation = 1, distribution symmetric). Regression models were used to adjust scores for the influence of age, sex, handedness, and robotic platform. Robotic platform was included as a covariate because data from each platform are on the same scale *within* each task, but note that behavioral *patterns across tasks* may vary. These equations were implemented in the software used to run the Kinarm tasks (Dexterit-E version 3.7; see text footnote 1) and generated *Z*-scores for measures of performance for each healthy individual. This process has been described in greater detail previously (BKIN Technologies, 2019; Simmatis et al., 2020a).

### Principal Component Analysis

Principal component analysis was conducted in R version 3.4.1 (R Core Team, 2017) using psych package version 1.8.12 (Revelle, 2019). Kinarm performance items were converted to Z-scores (Simmatis et al., 2020a) prior to PCA, with parallel analysis being used to determine the optimal number of components. Eigenvalues (i.e., proxies of relative variance explained by each component) were plotted in descending order to generate a scree plot (Cattell, 1966; Sakaluk and Short, 2017). Parallel analysis was used to generate a random data set that possessed the same dimensionality (i.e., number of columns and sample size) as the observed data. The 95th percentile (i.e., 1.645 standard deviations above the mean) of eigenvalues calculated from the randomized data was graphed over the scree plot of the observed data. The components retained from the observed data were those with larger eigenvalues than the corresponding random dataset (Horn, 1965; Cattell, 1966; O’connor, 2000; Sakaluk and Short, 2017). Parallel analysis was chosen as the method to retain components as the scree plot is only useful as an adjunct due to variability in accuracy, whereas parallel analysis has been shown to be highly robust to variation in data size/structure (Zwick and Velicer, 1986). See Supplementary Figure 1 for the scree plots and respective parallel analysis across tasks. Rotation was then implemented to help yield the most interpretable solution. An oblique rotation (oblimin) was first conducted (Jennrich and Sampson, 1966), which allows for correlated components. If all inter-component correlations were < | 0.30|, then an orthogonal rotation (varimax) (Kaiser, 1958) was implemented. Taking the current sample size into account, and using a two-tailed α of 0.01, we considered a component loading to be substantial if the loading was ≥ | 0.40| (Stevens, 2009). Note that for *n* = 300 a loading of 0.298 (2^∗^0.149) is considered substantial, we used ≥ |0.40| to keep in line with our previous work (Wood et al., 2018) and to remain stringent in identifying substantial loadings. Additionally, an item was considered to cross-load if the loading was ≥ | 0.30| on two or more components (Costello and Osborne, 2005). To further characterize performance, components and their respective parameters that loaded highly were used to generate intuitive labels. For complete details of the EP PCA, see our previously published findings (Wood et al., 2018). Although we considered all {1…*k; k* ≥ *n*} – component models, we describe in detail only the optimal *n*-component solution for the sake of brevity.

We retained the restricted analysis of right-handed individuals only from our previous work (Wood et al., 2018). We allowed individuals with left-hand dominance to be analyzed in the present study to maintain maximum comparability to our previous work and also ensure that we further expanded upon this previous limitation to now include both left- and right-handed participants. Importantly, the participants analyzed in the previous study had completed tasks with their non-dominant arms, but we chose to exclude these data for simplicity of interpretation of our results; our primary objective was the implementation of the PCA technique on granular robotic data. Furthermore, in the present work, we sought to expand the application of our technique to include non-dominant arm information.

### Determining PCA Agreement Across Platforms

We compared patterns of PC loadings after PC rotation by using the distance correlation metric (Székely et al., 2007). Briefly, the distance correlation is a tool for generalized correlation between series of data that are not necessarily linearly related. It calculates a Euclidean distance measure between two series of data (i.e., one set of PCs derived from each robot type) to determine how “close” they are in absolute terms without requiring specific data distributions (e.g., we would not expect PC values to be Normally distributed). Thus, we applied this technique to each pair of PC loadings to identify their similarity regardless of being in the same feature space due to rotation. Distance correlation is bounded between 0 and 1, with 0 indicating no agreement, and 1 indicating perfect agreement (Székely et al., 2007). We refer the interested reader to (Székely et al., 2007) for the in-depth calculation of the distance correlation and more detailed notes on its interpretation. Distance correlations and associated *p*-values (derived using a permutation method built into the software) were calculated using the Pingouin 0.3.8 package implemented in Python 3.7.1. Given the number of correlations performed (*n* = 61), we set the significant α value for correlations at 0.05/61 ≈ 8.19e-4 (i.e., we used the Bonferroni correction to control for the family-wise error rate).

## Results

### Data Reduction Related to the EXO

**FIGURE 2:**
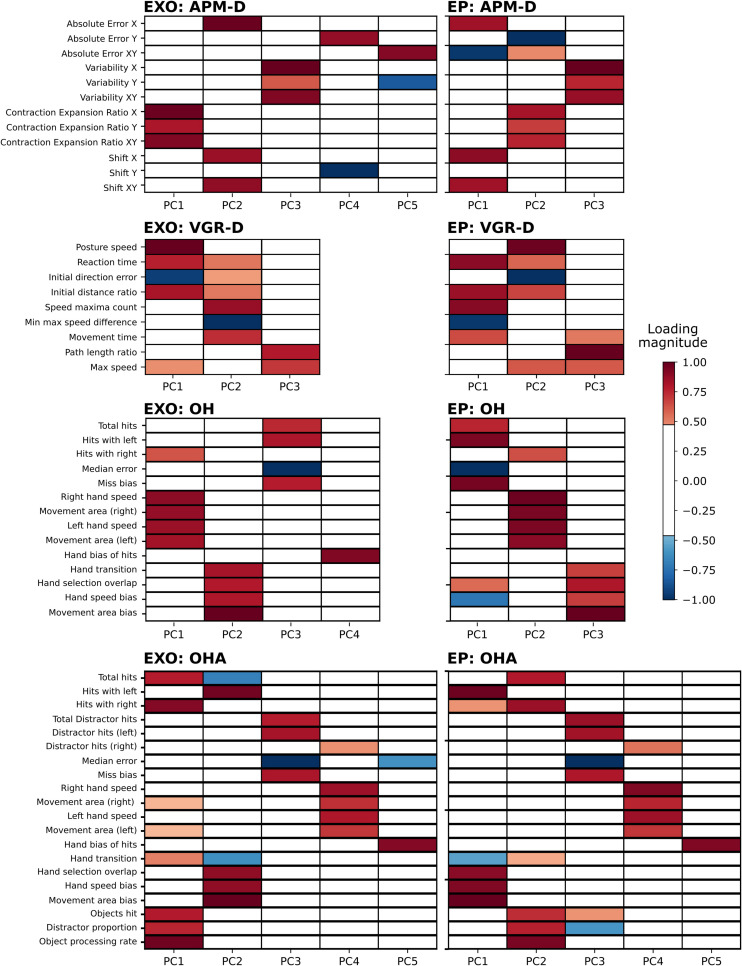
Heatmaps depicting substantial (>| 0.40|) loadings in the EXO and EP tasks. Results are summarized only for VGR and APM in the dominant arm, for comparison across robotic platforms (EP data do not include non-dominant arm analyses). **Left column**: EXO loadings. **Right column**: EP loadings. **Both columns**: highly positive loadings are indicated by red hues and highly negative loadings are indicated by blue hues. Loadings less than | 0.40| are white. “-D” signifies dominant arm.

Table 2 provides a brief summary of demographics for each cohort. Of all participants assessed using the EXO (*N* = 469), 211 (45%) were male, mean age was 46.4 (range: 18–93 years), and 418 (89%) were right-handed. Figure 2 displays the PC loadings for parameters of each task completed using the Kinarm EXO and EP platforms. The number of performance parameters per task (range 9–20) was substantially reduced (range 58–75%) by discovering groups of related parameters that gathered into 3–5 components, which explained a large amount of overall variance (range 76–87%) in each task. Although the ordering/variance accounted for by the PC varied across robotic platforms, the EXO PCs were comprised of several of the task parameters that loaded highly onto PCs derived from the EP data (i.e., consistent across platforms). For example, in APM (dominant arm), similar parameters that described EP variability (contraction/expansion ratio) had high loadings on the first component (all loadings >0.8) but several parameters loaded highly onto PC2 on the EP. Another example can be observed in OH, in which parameters related to either left- or right-hand speed, had moderate-to-high loadings on the first component (all >0.6). For complete details regarding the proportion of variance accounted for, data reduction, and component loadings, see Supplementary Tables 1, 2. The interpretation of the PCs derived from the EXO dataset was often similar to that for PCs from the EP dataset, and we have provided a summary of the interpretations of the top two PCs from each task across both platforms in Table 3.

**TABLE 2:** Participant demographics and task characteristics.

	**Task**	***N***	**Mean age (range)**	**Male sex (%)**	**Right-handedness (%)**
Exoskeleton	Arm Position Matching	469	46.39 (18–93)	211 (45%)	418 (89%)
	Visually Guided Reaching	469	46.39 (18–93)	211 (45%)	418 (89%)
	Object Hit	469	46.39 (18–93)	211 (45%)	418 (89%)
	Object Hit and Avoid	469	46.39 (18–93)	211 (45%)	418 (89%)
End-Point	Arm Position Matching	184	44.43 (18–87)	84 (46%)	184 (100%)
	Visually Guided Reaching	200	42.51 (18–88)	80 (40%)	200 (100%)
	Object Hit	190	46.28 (18–87)	86 (45%)	190 (100%)
	Object Hit and Avoid	170	45.56 (18–87)	76 (45%)	170 (100%)

**TABLE 3:** Top two PCs from each task and their interpretations.

**Platform**	**Task: component**	**Interpretation**
EXO	APM-D: PC1	*Contraction/expansion*: captures the extent that mirror-matched movements were horizontally (X)/vertically (Y)/both (XY) increased with respect to the movement of the active arm
	APM-D: PC2	*Error and shift*: captures the horizontal (X) error of the mirror-matched arm with respect to the active arm, as well as horizontal and vertical displacement (XY) with respect to the active arm
	VGR-D: PC1	*Initial movement*: parameters loading on this PC describe early phases of movement (e.g., reaction time and initial direction error)
	VGR-D: PC2	*Corrective-movements and total metrics*: this PC captures corrective features of reaching and total metrics, such as number of speed maxima, movement time, and difference between min and max speeds. Also captures some initial movement features
	OH: PC1	*Speed and area*: parameters associated with this PC describe hand speed and the amount of the workspace covered during the task
	OH: PC2	*Movement laterality*: this parameter captures the balance of use of both hands, e.g., hand selection overlap indicates how much of the workspace was shared by both hands’ movements during the task
	OHA: PC1	*Area and objects hit*: described both the ability to hit objects and the hitting of distractors (positive association), as well as area of movement throughout workspace (positive association)
	OHA: PC2	*Object hitting and laterality*: captured a negative association between total number of objects hit, and ability to use both hands to complete the task (e.g., hand selection overlap, which quantifies shared workspace area between both hands)
EP	APM-D: PC1	*Error and shift*: horizontal (X) and horizontal-vertical (XY) error measures and displacement with respect to the active arm
	APM-D: PC2	*Contraction/expansion*: horizontal (X), vertical (Y), and both (XY) stretching of the workspace of the participant’s mirror-matched movements with respect to the active arm’s target workspace. Also captures vertical and horizontal-vertical deviation from target locations
	VGR-D: PC1	*Corrective movements and total metrics*: parameters loaded onto this PC describe corrective aspects of reaching, such as speed maxima count, and total metrics like movement time
	VGR-D: PC2	*Initial movement*: this PC’s parameters emphasize the early phase of reaching, e.g., reaction time and initial distance ratio
	OH: PC1	*Goal-directed object hitting*: this PC described the ability to hit the virtual targets, both in terms of objects hit and also how long the participant could prolong being overwhelmed (median error)
	OH: PC2	*Speed and area*: this PC primarily captured left- and right-hand speed as well as the area that each hand covered in the workspace throughout the task
	OHA: PC1	*Hits and laterality*: primarily loaded by parameters describing the ability of both hands to contribute to the objective of hitting targets. For example, targets hit (both left and right hand) positively covaried with hand selection overlap
	OHA: PC2	*Object hitting*: captured both the ability to hit objects, as well as hitting of distractors (positive association between these)

### Agreement Between EXO and EP PCs

**FIGURE 3:**
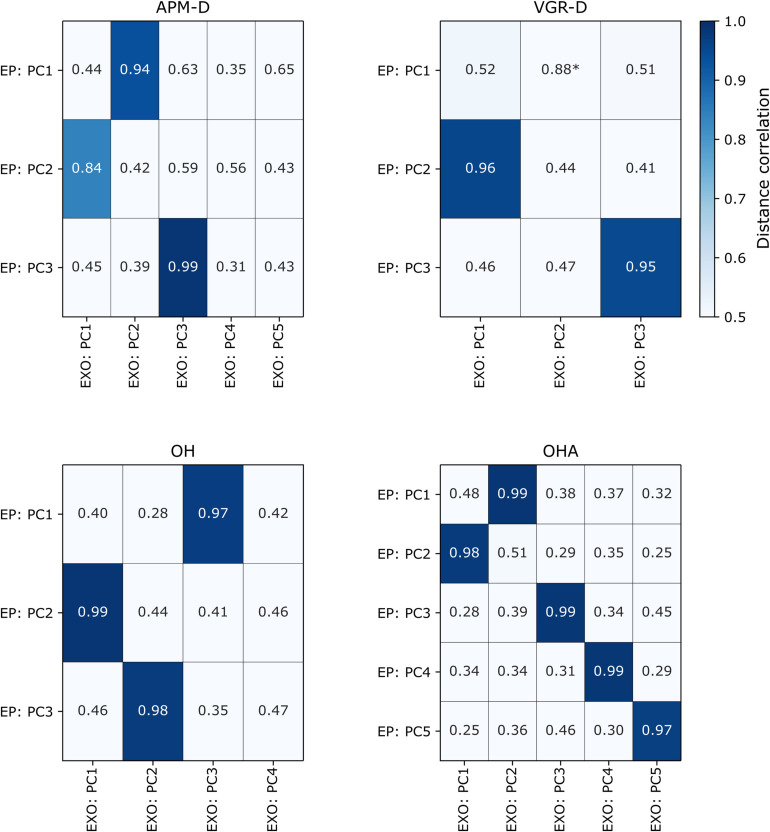
Distance correlations for each of the tasks in which comparison was possible between EXO and EP robots. Correlation values are colored darkening shades of blue if they are statistically significant after correcting for multiple comparisons (*p* < 8.19e-4), (i.e., lighter hues indicate lower correlation values). PC = “principal component”, “-D” = “dominant arm.” *Indicates correlations that were > 0.80 but had *p*-values < 0.05 but > 8.19e-4 (i.e., not statistically significant after multiple comparisons).

For the End-Point robot, *N* = 80/200 to 86/190 (40–46%) of participants were male, mean age was 42.5 (range 18–88) to 46.3 (range 18–87), and all participants for each task were right-handed. Note that this group included only right-handed individuals (Wood et al., 2018). We quantified agreement between EP and EXO robot PCs using the distance correlation, which were generally substantial (>0.50) (Figure 3). For tasks in which there were the same numbers of PCs across both analyses (i.e., VGR and OHA), each PC from the EXO robot had a clear correspondent in the EP data. For example, PC1 of VGR (EXO) had a distance correlation of 0.96 (*p* < 1e-4) with PC2 of VGR (EP), which is intuitive as PC2 of VGR (EP) was comprised of 6/8 of the task parameters that loaded highly onto PC1 of VGR (EXO). This PC agreement across platforms was most evident for the OHA task, where distance correlations across all five PCs were high (≥ 0.97; all *p*-values < 8.19e-4). However, other situations arose where there were differing numbers of components across platforms (i.e., APM and OH). Specifically, both OH and APM-D were represented by three PCs for the EP, but by more for the EXO (4 and 5, respectively). In these cases, high correlations (>0.80; all *p* < 8.19e-4) were still observed between the three PCs for the EP and one of the top three PCs for the EXO. Finally, we observed a case in VGR-D where a correlation was substantial (0.88) but not statistically significant (0.05 > *p* > 8.19e-4) between EP PC1 and EXO PC2.

### Principal Component Analysis Across All Four Kinarm Tasks

**FIGURE 4:**
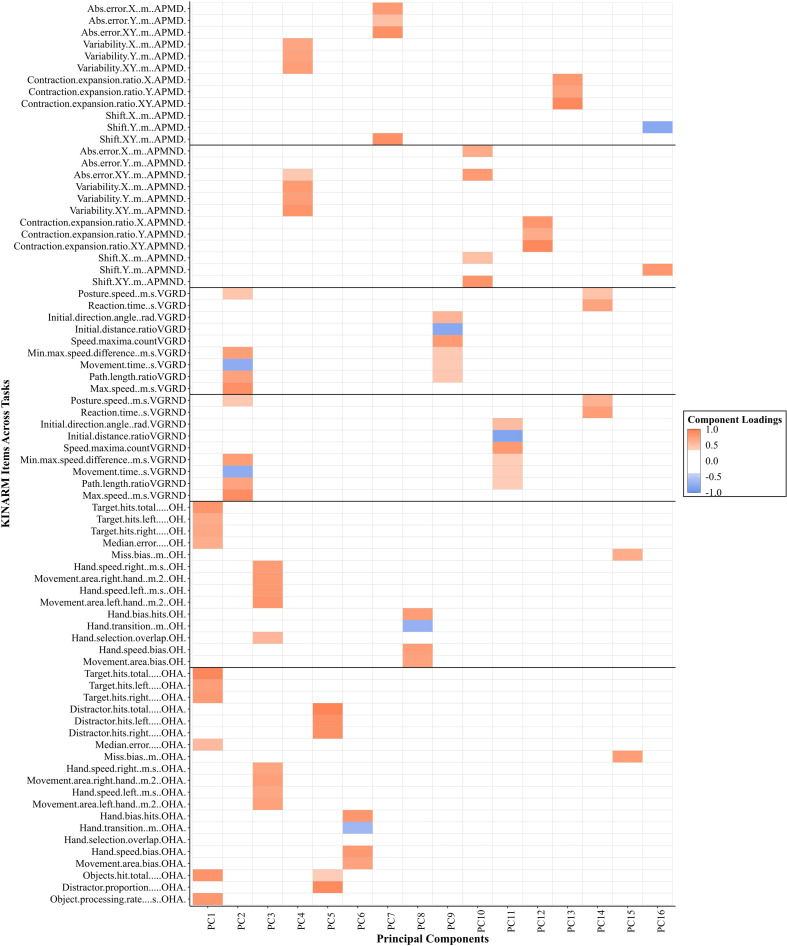
A visual representation of significant loadings (>| 0.40|) from the principal component analysis across four Kinarm tasks with all parameters included for healthy individuals. The color gradient represents the strength of the component loadings, with the darker shade representing higher loadings. Orange represents positive loadings with blue representing negative loadings. The gray lines visually separate tasks and the respective limb assessed. APM = Arm Position Matching; VGR = Visually Guided Reaching; RVGR = Reverse Visually Guided Reaching; OH = Object Hit; OHA = Object Hit and Avoid; D = dominant limb; ND = non-dominant limb.

Our final analysis examined if greater dimension reduction was possible if all the parameters across all behavioral tasks were considered simultaneously. According to the scree plot and parallel analysis, 16 models warranted further examination. For 2- through 16-component solutions, we kept the orthogonal rotation because the inter-component correlations ranged from −0.18 to 0.29. For the 16-component solution, all items had substantial loadings (>| 0.40|). See Figure 4 for a visual representation of the component loadings across tasks and respective items. When pooled together across all six tasks, PCA reduced this large data set from 76 parameters to 16 components, reducing the overall data by 79% while still retaining 73% of the variance. The across-task PCA identified fewer components than PCA on the individual tasks (16 PCs compared to a total of 24 PCs across individual tasks). Interestingly, each task generally remained separate on its own PC. Notably, both VGR and APM tasks commonly shared PCs across dominant and non-dominant arms. For example, PC2 was dominated by VGR across both arms. APM across both arms had several parameters that loaded onto PC4. As well, OH and OHA tasks also shared PCs (i.e., PC1 and PC3). In contrast, PCs never included OH or OHA parameters with VGR or APM parameters, and no PCs included VGR and APM parameters.

## Discussion

We performed PCA on Kinarm EXO data, similarly to our previous work with the Kinarm EP, to quantify the extent of agreement between the results of PCA on data collected from both platforms. We then performed PCA across multiple tasks on EXO data to investigate inter-task interactions between parameters. We identified that PCA of EXO data produced interpretable results that were subjectively comparable to our previous analysis. The strengths of the associations between results from both platforms suggest that participants performed behavioral tasks similarly with both the EP and the EXO systems with minimal, albeit informative, deviation of task parameter loadings across PCs and platforms. Finally, PCA across all tasks highlighted further dimensionality reduction beyond that observed in individual-task PCAs, reflecting interactions across tasks with similar characteristics. These results have interesting implications for the ability of different robotic platforms to quantify motor function, and for the use of robotic platforms to characterize motor impairments that may arise from neurological disease.

We identified that PCA of Kinarm EXO and EP data captured relationships between variables that were interpretable, and that differences across platforms could be emphasized using these findings. As an illustrative example of interpretability, consider the first component derived from VGR (dominant arm). Loadings on this component indicated that as total movement time decreased, maximum speed increased, as did the path length ratio (ratio of actual distance the hand traveled relative to the straight-line distance between start and end points). Intuitively, this group of variables would appear to be related as time and speed should be inversely related and longer trajectories generating a larger path length ratio should tend to require more time to complete the movement. Interpretability also extends to across-platform comparisons. Consider PC1 of OHA from the EXO, and PC2 of OHA from the EP (Figure 2), in which loaded parameters were largely the same except for “movement area” for both the right and left hand, which were uniquely observed with PC1 of the EXO. The covariation of these parameters with overall metrics of performance such as “total objects hit” suggests that, in the EXO platform, there was a stronger relationship between workspace utilization and overall task performance than there was with the EP. This difference likely reflects the fact that movement is less encumbered when using the EP as compared to the EXO robotic systems.

Our results illustrate that PCA on data gathered from the EP agreed very well with the PCA on data derived from the EXO versions of Kinarm robotic platforms; this demonstrates that some fundamental constructs of motor behavior were captured using both platforms. Similarity across platforms is most evident in VGR-D, where a three PC structure was chosen for both platforms; each component is largely comprised of the same task parameters and only their order differs, which is not surprising given that PC loadings are sample dependent and negligible differences will frequently be observed. Similarity in the parameters captured in a given PC also extends previous findings that arm movements can be performed similarly whether the elbow is free, or whether the entire arm is constrained to the horizontal plane while moving (Beer et al., 2004). The example of VGR-D has interesting implications from a motor control perspective. The three PCs that were found to describe VGR-D, for both platforms, had relevant groupings that correspond to features of motor behavior. PC1 from the EXO data describes initial movements, as does PC2 from EP data (see also Table 3). Interestingly, the parameters that loaded onto this component correspond to the early feedforward component of reaching (Coderre et al., 2010). This is in contrast to PC2 of EXO data and PC1 of EP data, which included parameters related to feedback control (Coderre et al., 2010). Although separating feedforward and feedback processes may not necessarily be correct (Todorov and Jordan, 2002; Crevecoeur and Kurtzer, 2018), there is a convenience in delineating early- and later epochs of movement. Further, the present results highlight that the strategies implemented by individuals tends to lead to distinct patterns in performance related to movement initiation and online corrections.

It is interesting to note the across-task PCA of EXO data demonstrated interactions within classes of tasks (i.e., reaching, hitting, and matching), but limited interactions between different classes of tasks. PCs spanned VGR across arms and APM across both arms, and OH and OHA, suggesting common strategies. In contrast, there was little shared information *between* the three classes of tasks. For example, speed in VGR and speed in OH/OHA did not group together. This separation in performance between motor tasks is consistent with previous work highlighting relatively low correlations in performance across different skills or tasks. For example, Drowatzky and Zuccato (1967) examined the performance of individuals for various static and dynamic postural tasks and found correlations were quite low (range from −0.19 to 0.31). As well, previous work demonstrated poor intra-individual correlations between movement speed and accuracy across unimanual- and bimanual upper-limb tasks when both hands have to reach to two targets simultaneously (Mickevičienė et al., 2015), although this may be different when there is a common goal for both hands (Tresilian and Stelmach, 1997). These findings suggest a goal-dependency of the separability of intra-individual unimanual and bimanual performance, which would additionally align with the differences in task objective between VGR and OH/OHA in the present study. As with the case for within-task PC loadings, across-task PC loading patterns have interesting implications from the perspective of the neural correlates of motor behavior. Uni- and bi-manual tasks are executed by the similar brain structures, but it is not the case that a bimanual movement is simply two yoked unimanual movements. For example, Grefkes (Grefkes et al., 2008) demonstrated that stroke can disrupt interhemispheric communication between supplementary motor area (SMA) and primary motor cortex (M1), which affected bimanual movements in a unique way compared to unimanual movements. Bimanual tasks recruit a distributed network of brain areas involved in movement control, including the bilateral dorsal premotor cortices and bilateral parietal association areas (Szameitat et al., 2012). Potentially, the PCs that we found for bimanual object-hitting tasks could provide insight into the functionality of these distributed networks. However, this is beyond the scope of our current analysis.

Our results suggest that it might be suitable to use Kinarm platforms interchangeably for clinical research, particularly that involving high-dimensional data analysis techniques. Using the example of VGR, it is clear that PC1 of the EXO and PC2 of the EP capture nearly an identical behavioral pattern. Platform interchangeability could potentially be important, for example, in individuals with multiple sclerosis (MS). People with MS can have wide-ranging levels of upper limb impairments (Simmatis et al., 2020b) and, as such, they may require the use of one machine or another, depending on their status. For less-affected individuals, it would be ideal to use an EP-style device to minimize setup time and mitigate fatigue-related effects induced by a long assessment. Conversely, in more-affected individuals who suffer from weakness or who readily experience motor fatigue, an EXO-style device could be used to provide upper-limb anti-gravity support. Importantly, the Z-scores derived from the Kinarm assessments are adjusted for age, sex, handedness, and robotic platform, and so it is valid to compare individual Z-scores regardless of platform. However, when considering analyses that might use high-dimensional behavioral data, such as multivariate statistics or machine learning to detect behavioral patterns, it will be important to consider the covariation of multiple parameters. In the example of VGR, because the individual parameters covary similarly across platforms (e.g., EXO PC1 and EP PC2), it would likely be suitable to put both EXO and EP data into one common analysis. Some tasks’ PCs displayed minor changes in loading magnitude (e.g., OHA EXO PC1 and OHA EP PC2 have similar but non-identical loading patterns), but likely not to the extent that it would confound multivariate analysis.

Our study has some limitations to address. First, our original assessment using EP data was restricted to right-handed individuals, and the non-dominant arm was not considered in analysis. Despite this, we identified very high levels of agreement between the tasks that we included previously and those analyzed here, and high agreement was consistent across tasks. Thus, it seems likely that assessments in the non-dominant limb, or in left-handed individuals, would yield comparable results to those observed here. Note also that our data analysis procedure adjusted for handedness in calculating normalized Z-scores. Given that we performed these assessments on healthy individuals, it remains to be seen how well these results generalize to individuals with neurological impairments and if PCA facilitates separation of healthy individuals and those with neurological deficits, such as stroke. It is possible that stroke perturbs normal motor synergies and therefore could disrupt the way that our kinematic parameters group together, which would be favorable if used as a preprocessing step for supervised classification. These problems need to be considered in future studies. Furthermore, there were differences in the number of participants in the study across both platforms, which may have contributed small differences in PC loadings or orderings.

## Conclusion

We identified that PCA of data from the Kinarm EXO was able to intuitively describe sensorimotor task performance, in line with our previous work using the Kinarm EP. We additionally identified across-task or across-limb associations of parameters that potentially highlight higher-level patterns of sensorimotor behavior in healthy individuals. Finally, we can quantitatively state that behavioral constructs uncovered using PCA on EP- and EXO robots were conserved across platforms. Future work should investigate the use of our PCA-based approach to improve the classification of clinical populations and assist with the characterization of neurological deficits.

## Data Availability Statement

The raw data supporting the conclusions of this article will be made available by the authors, without undue reservation.

## Ethics Statement

The studies involving human participants were reviewed and approved by the Queen’s University and Affiliated Hospitals Health Sciences Research Ethics Board University of Calgary’s Conjoint Health Research Ethics Board. The patients/participants provided their written informed consent to participate in this study.

## Author Contributions

MW, LS, and JJ participated in the statistical plan, data analysis, and drafting of the manuscript. SD participated in the study design, collection of the data, and drafting of the manuscript. JB participated in the study design and drafting of the manuscript. SS is the primary investigator and participated in the study design and drafting of the manuscript. All authors contributed to the article and approved the submitted version.

## Conflict of Interest

JB receives a stipend from the Trillium Gift of Life Network to support his role as the Hospital Donation Support Physician. SS is cofounder and CSO of Kinarm, the manufacturer of the robotic device used in the present study The remaining authors declare that the research was conducted in the absence of any commercial or financial relationships that could be construed as a potential conflict of interest.

## Footnotes

1 www.kinarm.com
