# Supplementary material for: Principal Components Analysis Using Data Collected From Healthy Individuals on Two Robotic Assessment Platforms Yields Similar Behavioral Patterns
Source: Front Hum Neurosci. 2021 May 6;15:652201. doi: 10.3389/fnhum.2021.652201 (PMC8134538; doi:10.3389/fnhum.2021.652201)
Supplement: Supplementary file 1 [file Table_1.DOCX]

Supplementary Material

# Supplementary Tables

| **Supplementary Table 1**. Proportion of variance explained by each component and percent of data reduction across KINARM tasks for healthy control participants. | | | | | | | | | | | | |
| --- | --- | --- | --- | --- | --- | --- | --- | --- | --- | --- | --- | --- |
| **Task** |  | **Number of Parameters Used for PCA** |  | **Variance explained per component** | | | | |  | **Cumulative**  **variance** |  | **% of metrics reduced by PCA** |
|  |  |  |  | 1 | 2 | 3 | 4 | 5 |  |  |  |  |
| APMD |  | 12 |  | 23% | 23% | 19% | 12% | 10% |  | 87% |  | 58% |
| APMND |  | 12 |  | 24% | 21% | 20% | 11% |  |  | 76% |  | 67% |
| VGRD |  | 9 |  | 34% | 30% | 14% |  |  |  | 78% |  | 67% |
| VGRND |  | 9 |  | 35% | 28% | 16% |  |  |  | 79% |  | 67% |
| OH |  | 14 |  | 25% | 22% | 20% | 9% |  |  | 76% |  | 71% |
| OHA |  | 20 |  | 22% | 20% | 16% | 15% | 7% |  | 80% |  | 75% |
| *Note*. Grey shaded region indicates that there are no components for these tasks. APMD: Arm Position Matching dominant limb; APMND: Arm Position Matching non-dominant limb; VGRD: Visually Guided Reaching dominant limb; VGRND: Visually Guided Reaching non-dominant limb; OH: Object Hit; OHA: Object Hit and Avoid. | | | | | | | | | | | | |

| **Supplementary Table 2.** Principal component loadings for the four KINARM tasks administered to healthy participants using the Exoskeleton robot. | | | | | | |
| --- | --- | --- | --- | --- | --- | --- |
|  |  | **Component Loadings** | | | | |
| **Task** | **Kinarm Items** | **1** | **2** | **3** | **4** | **5** |
|  |  |  |  |  |  |  |
| **Arm Position Matching**  **Dominant Limb** | Absolute Error X | 0.161 | **0.899** | -0.043 | 0.193 | -0.050 |
|  | Absolute Error Y | 0.171 | 0.256 | -0.019 | **-0.689** | 0.054 |
|  | Absolute Error XY | 0.163 | **0.874** | -0.039 | -0.134 | -0.017 |
|  | Variability X | **0.927** | 0.036 | 0.050 | 0.044 | 0.054 |
|  | Variability Y | **0.836** | -0.040 | -0.086 | -0.101 | -0.073 |
|  | Variability XY | **0.977** | 0.023 | 0.018 | 0.008 | 0.032 |
|  | Contraction Expansion Ratio X | -0.033 | 0.045 | **0.930** | 0.058 | 0.260 |
|  | Contraction Expansion Ratio Y | 0.093 | -0.100 | **0.668** | -0.110 | **-0.554** |
|  | Contraction Expansion Ratio XY | 0.013 | -0.018 | **0.991** | 0.007 | -0.053 |
|  | Shift X | 0.054 | -0.103 | 0.116 | -0.045 | **0.923** |
|  | Shift Y | 0.053 | 0.054 | 0.024 | **0.889** | -0.013 |
|  | Shift XY | -0.191 | **0.979** | 0.058 | -0.104 | -0.012 |
|  |  |  |  |  |  |  |
| **Arm Position Matching**  **Non-Dominant Limb** | Absolute Error X | 0.183 | **0.637** | -0.103 | 0.090 |  |
|  | Absolute Error Y | 0.263 | **0.411** | 0.057 | **-0.521** |  |
|  | Absolute Error XY | 0.277 | **0.826** | -0.056 | -0.063 |  |
|  | Variability X | **0.920** | 0.031 | 0.038 | 0.103 |  |
|  | Variability Y | **0.840** | -0.035 | -0.039 | -0.188 |  |
|  | Variability XY | **0.969** | 0.017 | 0.025 | 0.048 |  |
|  | Contraction Expansion Ratio X | 0.039 | 0.027 | **0.887** | 0.247 |  |
|  | Contraction Expansion Ratio Y | -0.020 | -0.111 | **0.712** | **-0.443** |  |
|  | Contraction Expansion Ratio XY | 0.010 | -0.027 | **0.992** | -0.030 |  |
|  | Shift X | 0.010 | 0.372 | 0.213 | **0.663** |  |
|  | Shift Y | 0.185 | -0.290 | -0.079 | **0.572** |  |
|  | Shift XY | -0.163 | **0.960** | -0.008 | 0.028 |  |
|  |  |  |  |  |  |  |
| **Visually Guided Reaching Dominant**  **Limb** | Posture speed | **0.446** | -0.016 | **0.673** |  |  |
|  | Reaction time | -0.310 | 0.102 | **0.756** |  |  |
|  | Initial direction error | 0.179 | **0.702** | 0.274 |  |  |
|  | Initial distance ratio | 0.066 | **-0.897** | -0.144 |  |  |
|  | Speed maxima count | -0.095 | **0.824** | -0.261 |  |  |
|  | Min max speed difference | **0.780** | **0.504** | 0.031 |  |  |
|  | Movement time | **-0.840** | **0.412** | -0.135 |  |  |
|  | Path length ratio | **0.735** | **0.516** | -0.050 |  |  |
|  | Max speed | **0.940** | -0.098 | -0.093 |  |  |
|  |  |  |  |  |  |  |
| **Visually Guided Reaching**  **Non-Dominant**  **Limb** | Posture speed | **0.429** | -0.034 | **0.675** |  |  |
|  | Reaction time | -0.269 | 0.030 | **0.811** |  |  |
|  | Initial direction error | 0.300 | **0.623** | 0.398 |  |  |
|  | Initial distance ratio | 0.004 | **-0.899** | -0.135 |  |  |
|  | Speed maxima count | -0.070 | **0.823** | -0.320 |  |  |
|  | Min max speed difference | **0.812** | **0.450** | 0.099 |  |  |
|  | Movement time | **-0.791** | **0.482** | -0.141 |  |  |
|  | Path length ratio | **0.771** | **0.459** | 0.009 |  |  |
|  | Max speed | **0.937** | -0.135 | -0.096 |  |  |
|  |  |  |  |  |  |  |
| **Object Hit** | Total hits | 0.024 | **0.975** | 0.002 | 0.006 |  |
|  | Hits with left | -0.093 | **0.804** | -0.323 | 0.258 |  |
|  | Hits with right | 0.107 | **0.798** | 0.329 | -0.262 |  |
|  | Median error | -0.050 | **0.827** | -0.056 | -0.064 |  |
|  | Miss bias | 0.022 | -0.049 | 0.108 | **0.913** |  |
|  | Right hand speed | **0.838** | 0.177 | 0.231 | -0.096 |  |
|  | Movement area (right) | **0.852** | -0.064 | 0.264 | 0.043 |  |
|  | Left hand speed | **0.869** | 0.132 | -0.196 | 0.040 |  |
|  | Movement area (left) | **0.881** | -0.059 | -0.147 | -0.071 |  |
|  | Hand bias of hits | 0.170 | -0.099 | **0.786** | -0.369 |  |
|  | Hand transition | -0.077 | 0.053 | **-0.777** | -0.307 |  |
|  | Hand selection overlap | **0.648** | -0.152 | 0.032 | 0.053 |  |
|  | Hand speed bias | -0.053 | 0.046 | **0.813** | -0.108 |  |
|  | Movement area bias | -0.028 | 0.002 | **0.761** | 0.235 |  |
|  |  |  |  |  |  |  |
|  |  |  |  |  |  |  |
| **Object Hit and Avoid** | Total hits | **0.964** | -0.168 | -0.013 | 0.111 | 0.013 |
|  | Hits with left | **0.782** | -0.158 | -0.358 | 0.048 | 0.246 |
|  | Hits with right | **0.809** | -0.109 | 0.338 | 0.143 | -0.215 |
|  | Total Distractor hits | -0.063 | **0.988** | 0.027 | 0.082 | 0.019 |
|  | Distractor hits (left) | -0.053 | **0.889** | -0.002 | 0.066 | 0.100 |
|  | Distractor hits (right) | -0.049 | **0.890** | 0.051 | 0.074 | -0.074 |
|  | Median error | **0.557** | **-0.419** | -0.014 | -0.123 | -0.006 |
|  | Miss bias | 0.027 | 0.039 | 0.006 | 0.006 | **0.919** |
|  | Right hand speed | **0.408** | 0.185 | 0.244 | **0.736** | -0.070 |
|  | Movement area (right) | 0.069 | 0.050 | 0.333 | **0.820** | 0.103 |
|  | Left hand speed | **0.410** | 0.142 | -0.253 | **0.755** | 0.115 |
|  | Movement area (left) | 0.100 | 0.044 | -0.201 | **0.865** | -0.117 |
|  | Hand bias of hits | 0.030 | 0.023 | **0.826** | 0.065 | -0.367 |
|  | Hand transition | -0.004 | -0.034 | **-0.753** | -0.085 | **-0.408** |
|  | Hand selection overlap | -0.206 | 0.003 | -0.031 | **0.517** | 0.008 |
|  | Hand speed bias | -0.011 | 0.037 | **0.843** | -0.045 | -0.201 |
|  | Movement area bias | -0.052 | 0.015 | **0.802** | -0.076 | 0.289 |
|  | Objects hit | **0.917** | 0.304 | -0.009 | 0.153 | 0.014 |
|  | Distractor proportion | -0.250 | **0.957** | 0.028 | 0.049 | 0.013 |
|  | Object processing rate | **0.804** | **-0.470** | -0.027 | 0.046 | 0.007 |
| *Note.* Bold text indicates that the component loading is substantial (≥ \|0.40\|). | | | | | | |

# Supplementary Figure
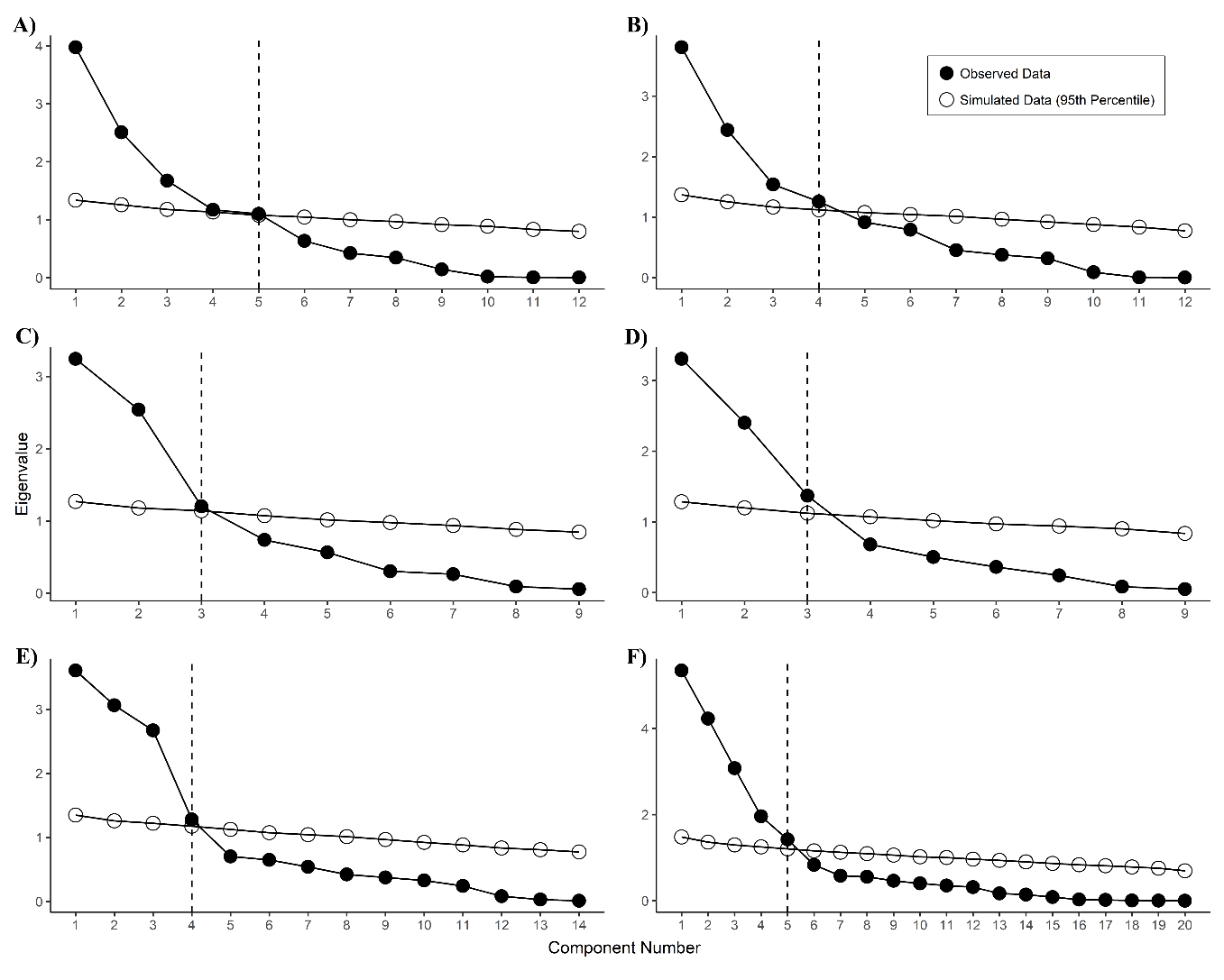


**Supplementary Figure 1.** Scree plots with parallel analysis indicating the number of components to be selected per Kinarm tasks for healthy controls. Dashed lines represent the number of components with eigenvalues that exceed those of the 95^th^ percentile of randomly generated eigenvalues (i.e. 1.645 standard deviations above the mean of the random eigenvalues). White markers represent the 95^th^ percentiles of randomly generated eigenvalues, and black markers represent real eigenvalues generated from Kinarm data. A) Arm Position Matching Dominant Limb; B) Arm Position Matching Non-Dominant Limb; C) Visually Guided Reaching Dominant Limb; D) Visually Guided Reaching Non-Dominant Limb; E) Object Hit; F) Object Hit and Avoid.
